# Water in protein hydration and ligand recognition

**DOI:** 10.1002/jmr.2810

**Published:** 2019-08-27

**Authors:** Manuela Maurer, Chris Oostenbrink

**Affiliations:** ^1^ Institute of Molecular Modeling and Simulation University of Natural Resources and Life Sciences Vienna Austria

**Keywords:** drug design, enthalpy entropy compensation, hydrogen bonds, water bridges, water structure

## Abstract

This review describes selected basics of water in biomolecular recognition. We focus on a qualitative understanding of the most important physical aspects, how these change in magnitude between bulk water and protein environment, and how the roles that water plays for proteins arise from them. These roles include mechanical support, thermal coupling, dielectric screening, mass and charge transport, and the competition with a ligand for the occupation of a binding site. The presence or absence of water has ramifications that range from the thermodynamic binding signature of a single ligand up to cellular survival. The large inhomogeneity in water density, polarity and mobility around a solute is hard to assess in experiment. This is a source of many difficulties in the solvation of protein models and computational studies that attempt to elucidate or predict ligand recognition. The influence of water in a protein binding site on the experimental enthalpic and entropic signature of ligand binding is still a point of much debate. The strong water‐water interaction in enthalpic terms is counteracted by a water molecule's high mobility in entropic terms. The complete arrest of a water molecule's mobility sets a limit on the entropic contribution of a water displacement process, while the solvent environment sets limits on ligand reactivity.

## Graphical abstract

This review describes selected basics of water in biomolecular recognition. We focus on aqualitative understanding of the most important physical aspects, how these change between bulk water and protein environment, and how they influence the roles that water plays for protein stability and protein‐ligand interactions.

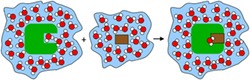

## 1 INTRODUCTION

Water and hydrogen bonding have been intensely investigated for a long time. They are still, to this day, the subject of wide‐ranging research and surprisingly heated debates. We suggest Pauling^1^ and Gilli^2^ for the basics of hydrogen bonding, Arunan^3^ for its current IUPAC definition, Biedermannová and Schneider^4^ and Bellissent‐Funel et al^5^ for methodological reviews on protein hydration, and Spyrakis et al^6^ for the application to ligand design. The popularity of water and hydrogen bonding in research is not only due to their central importance for life but also because they represent a conceptually simple yet arbitrarily challenging benchmark system for many approaches and applications. Water is the most investigated liquid in scientific literature, and any review of it must hence remain exemplary.

The isolated water molecule and the hydrogen‐bonded water dimer are relatively well understood meanwhile.^7^, ^8^ Current research focuses mainly on the complex effects that occur in larger agglomerates, and on the emergence of bulk properties when the number of water molecules is successively increased. Small water clusters are often investigated without a confining material structure such as a protein binding pocket. However, one could consider vacuum as the ideal hydrophobic surface—see Shin and Willard^9^ for a recent systematic exploration. Therefore, we begin with small water clusters and their unperturbed properties in vacuo as a model before turning to water within the confines of a binding pocket.

Prominent experimental methods include X‐ray and neutron scattering,^10^ nuclear magnetic resonance (NMR),^11^, ^12^ terahertz spectroscopy,^13^, ^14^, ^15^ femtosecond,^16^ infrared (IR),^17^ and two‐dimensional IR experiments^18^, ^19^, ^20^, ^21^ and Raman spectroscopy.^15^, ^22^, ^23^ Water research is surprisingly challenging, whether it is the interpretation of bulk spectroscopic data or the preparation of water samples with a defined number of molecules in vacuum.^17^ In the context of protein‐ligand recognition, only small numbers of water molecules are relevant since the amount that can be expected to occur in the active site of a protein is rather limited, as will be described in more detail below. Similarly, the number of water molecules that might be targeted for replacement by an additional functional group during ligand design is usually restricted to one, or a few at most.

Prominent computational methods used to study water and hydrogen bonding include the quantum theory of atoms in molecules (QTAIM^24^, ^25^, ^26^) and the natural bond orbital analysis, 27, 28 energy decomposition analysis,^29^, ^30^ density functional theory,^31^, ^32^ and molecular dynamics (MD) simulations,^9^, ^20^, ^33^, ^34^, ^35^, ^36^, ^37^ which are often also needed to interpret spectroscopy results.^15^, ^16^, ^22^ The clusters that are computationally investigated to very high accuracy usually still consist of only 2 to 50 water molecules at once,^37^ since calculations at this level of theory are computationally quite expensive.

Overall, this work does not aim to give a complete overview of experimental or computational methods. Rather, examples are selected to represent the basic principles of water in interaction with biomolecules. Bulk water properties are not the focus of interest here, but as they emerge naturally from the smaller units, and because they provide a frame of reference, mentioning them can hardly be avoided. We start with some current research on the basics that give rise to the familiar bulk picture of water in the macroscopic limit. We then take a look at general effects of a solute, followed by specific aspects of protein‐water interactions.

### 1.1 Structural properties

#### 1.1.1 Hydrogen bond strength and cooperativity

A key aspect is the strength of the hydrogen bond (H‐bond). It is often expressed either in terms of the stretching force constant (local stretching vibration),^38^ the bond dissociation energy,^39^ or the bond strength order.^40^ It depends on the donor and acceptor atoms^41^ as well as on the field generated by the surrounding atoms.

The strength of an H‐bond between water molecules can be considered to start at about 3 kJ mol^‐1^, in terms of potential energy, which is just about enough to overcome the 2.5 kJ mol^‐1^ average thermal energy at room temperature. The average liquid water H‐bond has about 15‐20 kJ mol^‐1^ of potential energy and 1.8‐2 Å length.^42^, ^43^, ^44^ That is about 5% of the strength and double the distance of the covalent oxygen‐hydrogen bond.^39^ In terms of bond strength order, this compares as 0.4 to 1.^37^ The upper limit on strength seems to be around 30 kJ mol^‐1^ nm^‐2^ in terms of stretching force constant; this is realized only when peripheral H‐bonded water molecules influence the central ones in an ideal arrangement. This push‐pull^45^ or cooperativity effect^46^ means that in a chain of H‐bonded water molecules, the first bond is the hardest to break, and successive ones become easier. Cooperative, or nonpairwise‐additive, intermolecular forces have been estimated to account for up to 25% of the cohesive energy of bulk water.^47^, ^48^ They are caused by charge transfer (CT) between donor and acceptor atoms, which increases the covalent character of the otherwise mainly electrostatic attraction between the dipoles.^2^, ^37^, ^45^, ^49^, ^50^

Due to the H‐bond's mixed and flexible nature, its bond strength is less connected to the angle and interatomic distance than in the case of covalent bonds.^37^, ^40^ This makes the H‐bond's properties more individualistic and more difficult to predict.^2^ Therefore, its very definition becomes somewhat arbitrary,^23^, ^44^ which causes a lot of variation between calculations, experiments, and the conclusions drawn from them. For example, the covalent part of the H‐bond has been a matter of recurring controversy^46^, ^49^, ^50^, ^51^ since its original estimate by Pauling at about 10% in terms of energy.^1^ This original rough estimate holds qualitatively true in the face of several modern quantum chemical analyses.^28^, ^52^ Widely varying estimates can still be found (some up to 40% or more^53^), but at least only the extent of the covalence, and not its existence, are debated meanwhile.^54^ The above‐mentioned theoretical approaches have been supported by various experimental ones^55^, ^56^ including, for example, an improved variant of atomic force microscopy with atomic bond resolution.^57^

#### 1.1.2 From clusters to bulk

Bulk water's pronounced and important ability to solvate polar molecules stems from its asymmetric charge distribution. The isolated molecule has a dipole moment μ of about 6 × 10^‐30^ C m^‐1^, or 1.85 Debye (D). The lone pair electrons on the oxygen atom that are the main cause for this uneven charge distribution are relatively mobile and displaceable by an external electric field. The molecular polarizability α describes the degree of responsivity of a molecule to an electric field. A single water molecule in vacuum has an experimentally measured polarizability of 1.6 × 10^‐37^ C^2^ m^2^ kJ^‐1^, or 9.9 atomic units (au).^58^

Two H‐bonded water molecules increase their dipole moments above the value expected from simple electrostatic considerations because CT along the H‐bond moves the involved lone pair electrons exceptionally far away from the oxygen atom. This distance allows for an angular decrease between the H‐bonding lone pair and the remaining one.^59^ In the asymmetric hydrogen‐bonded equilibrium geometry of a water dimer, the dipole moments of the donor and acceptor water molecules amount to about 2.09 D and 2.16 D, respectively.^60^ The net dipole moment has experimentally been determined to be 2.64 D for the dimer.^61^ However, the more localized an electronic charge becomes, the less it can be influenced by an electric field. This reduces the polarizability of a water molecule in the dimer by almost 20% in the bond direction.^60^ The average polarizability is reduced to about 7.8 au for the donor and 7.5 au for the acceptor molecule.^60^ All values for cluster polarizability rely on calculations because it seems that experimental confirmation is still not possible.^62^

The possible number of ideal H‐bonded geometries increases exponentially with the available number of molecules.^63^ For seven water molecules, there are already about 160 distinct configurations to consider, for 10 molecules it is about 1800.^64^ In general, more molecules lead to larger changes from the monomer and stronger bonds.^59^, ^65^

The water trimer is the easiest system in which the cooperativity of H‐bonding can be studied, and the three‐body‐interaction is estimated to contribute about 20% to the total trimer stability.^47^ The two basic arrangements are a linear geometry or a circular one. While the circular geometry is rather fixed and slightly strained, the molecules in a linear arrangement have more freedom of movement. In the circular case, the additional bond yields about 4 kJ mol^‐1^ more stability in terms of potential energy.^48^ However, for the water trimer at ambient conditions, the linear conformation seems just slightly more favorable in terms of free energy.^65^

For the tetramer and pentamer, the quasi‐planar cyclic structures are more stable.^64^ They assume the same geometries as their covalently connected carbon analogs in the absence of a confining structure. Well‐designed confinement can allow water molecules to take the same energetically favorable positions as they would by themselves; the water pentamer in the mostly hydrophobic biotin‐binding site of streptavidin seems an important example of this.^66^

The minimum potential‐energy structure of the water hexamer is the cage‐like three‐dimensional array found in ice (*I*_*h*_) with a measured net dipole moment of 1.85 D.^61^ In terms of free energy, the three‐dimensional arrays are only marginally less stable at room temperature than the quasi‐planar cyclic hexamer.^67^ From the hexamer on, prism or cage type structures are favored in this regard. As a general rule, lower temperatures and larger populations favour four‐coordinated molecules; and higher temperatures and smaller populations favour two‐coordinated molecules.^67^

Given a suitable static arrangement, it seems that the covalent character of the H‐bond overtakes the dipolar one somewhere between four and six water molecules.^45^ The dipole moment of a water molecule in a cluster increases strongly with the number of molecules due to cooperativity, until the curve begins to level off at about 2.76 D with the hexamer.^61^ The same flattening trend can be observed for the intermolecular distance.^61^ The molecular polarizability of the quasi‐planar cyclic hexamer amounts to only about 7 au,^68^ but in the three‐dimensional hexamers, it can decrease even further to 6.2 au.^69^

With growing cluster size, the molecular polarizability of water converges to an estimated bulk value between 4 and 5 au.^62^ The estimated average dipole moment of a water molecule in bulk is between 3 and 3.5 D.^59^, ^70^, ^71^ In the macroscopic limit, the net dipole moment over all water molecules in the liquid bulk phase is bound to cancel out statistically. The mean‐square fluctuations of the net dipole moment, however, are not zero.^72^ They are proportional to the dielectric constant, or relative (to vacuum) permittivity, ε_r_.^73^, ^74^ Bulk water has a relative dielectric constant of 78 at 25°C.

### 1.2 Disturbances

#### 1.2.1 The disordered network

Thermal motion introduces a change from all‐ideal geometries and one single type of H‐bond to a distribution of positions and orientations, and consequently the occurrence of weakened or unfulfilled H‐bonds. This distorted network still seems to involve somewhere between 90%^75^ and 98%^37^ of the water molecules in a sample at room temperature, though its extent has been wildly debated.^22^, ^76^ It transfers perturbations, such as excess energy input, and distributes it into the bulk through coupled modes of motion.^18^, ^21^ The order‐disorder dynamics of water span several orders of magnitude in molecular distances and timescales.^21^ The nature of liquid bulk water is highly complicated, and its structure has not been fully described to this day. Many attempts have been made to model bulk water properties as a “cluster of clusters.”^67^ Such models, in one way or another, stack equilibrium structures of water clusters from vacuum or confinement to build up a bulk phase model.^77^ Here, it shall suffice to say that these attempts were met with only modest success^63^ and were at times even labeled as “unconstructive.”^78^

In any case, atomic positions and distance‐dependent disorder are relatively simple parameters and accessible in scattering experiments. They are related to macroscopic bulk density, and the radial distribution function of a computational water model can be compared with it, for example.

The dynamics of water are more complex, however, and involve several different modes of motion. A basic property is the self‐diffusion coefficient, which is 2.3 × 10^‐5^ cm^2^ s^‐1^.^79^ The most important motion involved in breaking an H‐bond in the network is the hindered rotation, or libration.^80^ Librations are responsible for the ultrafast loss of structural “memory” (that is, the decay of correlation of a parameter over time) 21: The characteristic reorientation time for a water molecule in the bulk phase, and thus the life time of an H‐bond, is only on the order of ~2 ps.^13^, ^21^, ^80^

#### 1.2.2 Interfaces

As the orientation of each water molecule changes due to thermal motion, so does its dipole moment vector.^73^ Both locally and macroscopically, the net dipole moment can become different from zero. The bulk response to a macroscopic electric field (χ_e_) can be related to the molecular scale by accounting for the possible change in both microscopic dipole orientation and magnitude, countering the incident field.^73^ In addition to the effect of a local electric field, H‐bond dynamics also restrict possible changes in orientation. A difference in the properties and structure of water next to an interface will lead to a local difference in, eg, charge mobility, diffusive transport, or solubility. One of the smallest limitations that can be introduced to liquid bulk water is a single, at least partially hydrophobic solute molecule, consisting only of a few atoms.

It has often been observed that the H‐bond network around a hydrophobic solute is more rigid and distorted compared with bulk.^76^ The network must warp around the perturbation the solute represents, so the average tetrahedral coordination geometry gets distorted in its vicinity. The geometric constraint imposed by such an excluded volume prevents the fulfilment of some H‐bond possibilities. Accordingly, water molecules experience more attraction from the direction that provides more H‐bond possibilities, that is, the bulk.^9^, ^81^ They become preferentially oriented and positioned towards it and away from the solute. The solvation shell thus becomes locally anisotropic. The resulting gap between a hydrophobic solute and water is basically the same for a hydrophobic surface, a gas phase interface, or vacuum, and this is sometimes termed the dewetting effect.^82^ The number of unfulfilled or dangling H‐bonds is a matter of ongoing debate, probably in part due to the different physicochemical nature of different employed solutes.

The water molecules next to an excluded volume are limited not only in their optimal spatial positioning but also in their overall movement possibilities. Mobility necessarily slows down in vicinity of a solute.^13^, ^83^ This decreased mobility means that already existing H‐bonding partners are preferentially kept instead of swapped, and their orientation remains more constant. Thus, mutual adjustment is more pronounced, so the number of strong water H‐bonds increases at the expense of weaker ones around a solute.^13^, ^81^ This means the distribution of possible H‐bond energies narrows. Although the geometry is distorted from bulk, this represents an increase in molecular order: an unfavourable decrease in entropy for the water molecules in a solvation shell that is induced by a favourable enthalpic interaction between them.

Assuming a constant total solute volume, more ordered water molecules are required to build the hydration shell around two small solutes compared with one big one, due to its smaller surface. By merging two excluded volumes, the overall ordering in the system decreases, since more water molecules are free to switch between different bond strengths and partners. This is the assumed cause for the entropically favourable thermodynamic signature of hydrophobic association.^18^, ^84^, ^85^ Like the nature of bulk water, solvation is hotly debated, and cannot be fully described to this day. Disturbances specific to protein solutes will follow in the next chapter.

#### 1.2.3 Confinement

Geometric distortion and kinetic inhibition increase with the amount of excluded volume. Conceptually, this progresses with contact curvature in the order small solute—flat surface/interface—deep cavity/confinement. It is widely accepted that the presence of an excluded volume has a larger influence on dynamics than its chemical nature.^81^, ^86^ The most extreme case is, of course, solitary confinement—a case that can potentially occur often in proteins (see Section 2.2).

The loss of translational and rotational degrees of freedom in confinement means a loss in a water molecule's configurational entropy. The maximum entropic cost has been estimated by comparison with inorganic salt hydrates, where water is very strongly bound.^87^ For a single water molecule, it amounts to up to 28 J mol^‐1^ K^‐1^. At room temperature, this results in a potential maximum contribution to the free energy of 8 kJ mol^‐1^ for any process that releases trapped water from a confining environment.

## 2 PROTEIN HYDRATION

### 2.1 Proteins in water

#### 2.1.1 Intracellular hydration

Proteins operate in the crowded aqueous medium of living cells. Water supply is obviously crucial for a healthy organism, but the extent differs between species, and also between tissues. Under ideal conditions, the mass fraction of water in a cell ranges from the usual ~70% for, for example, human red blood cell or *Escherichia coli*, down to ~40% for *Bacillus subtilis* spores, which can endure extreme levels of heat, radiation, and chemicals.^88^ Some *tardigrada*, claimed to be the overall most resilient species on earth, can be dessicated to ~3% water content under slow drying conditions and still be revived.

Proteins constitute the majority of dry mass of a cell. Low water content is, like low temperature, associated with long‐term biopreservation through the suppression of protein movement.^89^ An additional side effect is that chemical denaturants, such as reactive oxygen species, are restricted from reaching the protein through the solvent (cytoplasm) under such conditions.

In addition to their mobility, proteins quickly lose their structure and function when they become dehydrated. This is in contrast to some other functional materials, for example, simple inorganic catalysts. The functionality, however, is part of the protein, not the water; therefore, one could consider water as a cofactor. In general, a water content of ~0.2 g water per g protein, which corresponds to less than one hydration layer, seems to be a relatively sharp necessary minimum.^4^ Once the critical hydration level is surpassed, a protein functions almost normally.

Only the proteins of extreme halophiles such as *Haloarcula marismortui* are natively surrounded by such a low amount of hydration. These halophiles compensate the lacking number of water molecules with the addition of about half again as many salt ions.^90^ This serves to stabilize the highly acidic protein structures that are evolutionarily adapted to minimize their solvent‐accessible surface area (SASA).^91^ Such a low a water content is not viable for the rest of the cell, and not even *Bacillus subtilis* operates around this level—spores are a dormant state. In general, organisms that can outlast desiccation or cold need time to produce protective molecules, which change the cell water structure or replace cell water entirely.

In a cell with a more common water content of around 6 g per g protein, only about three or four layers of water molecules are available between macromolecules.^92^ Water within a cell is therefore not considered to be bulk‐like, since it is known that the presence of solutes disturbs the H‐bond network for several layers. Water becomes structured differently in the field of a protein: It has been found to be about 15% more dense on the surface of a protein than in the bulk phase.^4^, ^93^, ^94^, ^95^ The cellular mixture results in a macroscopic viscosity of the cytoplasm that is similar to a gel, about ~10^6^ times higher than pure bulk water.^88^

It is thus perhaps surprising that the microscopic picture of cellular water is much more free‐flowing than the macroscopic one. In general, different measurement methods agree that most of the cytoplasmic water in a cell shows a dynamic behaviour similar to that of bulk water or salt solutions,^96^ regarded as necessary for osmotic activity. Less than half of cellular water is categorized as “slow water,” retarded in its dynamics by one to three orders of magnitude.

The maximum amount of “slow water” has been measured in *Haloarcula marismortui* by H‐NMR to be ~75%, but it was concluded that neither its proteins nor the high salt concentrations alone could account for this retardation.^90^ Most estimates range from ~10 to 20% of all cellular water in human red blood cells^12^ and *Escherichia coli* 88, 97 to ~45% in *Bacillus subtilis* spores.^88^ The calculated amounts differ since some experiments probe collective motions, while others probe individual molecules, and also assumptions for interpretation can vary.^93^ Slow water is strongly bound to biomacromolecules or trapped in the recesses of larger complexes. It is thought to bind mainly to the surfaces, and little is expected to be buried.^88^ Water can also be distributed inhomogeneously in cellular compartments.

#### 2.1.2 Water around proteins

The dilute situation in the majority of experiments is quite different from the crowded situation in a living cell. A working definition of hydration water is also important for biomolecular simulations. The magnitude as well as spatial extent of the perturbation a protein introduces on the surrounding water structure is still strongly debated, despite much research,^4^, ^93^ and results differ for the aforementioned reasons (experimental time frame, probed motions, energy thresholds, etc.). In principle, any change relative to bulk can be considered a perturbation, but the magnitude and implications are different. The experimental ~15% increase in density around a protein persists only for approximately the first water layer.^93^ It is caused by the disorder on the protein surface, which prevents the formation of a regular ice lattice and thus lowers the freezing point around a protein.^13^

Generally, individual properties are less affected by the excluded volume effect (see Section 1.2) than collective ones.^12^, ^93^ It seems that translation as measured by water's diffusion coefficient is more homogeneously affected than rotation, as measured by water's dipole orientation, at least on the protein surface.^12^ Translation is mainly retarded perpendicular to the surface, by about a factor of 3^13^, ^93^, ^98^ but not far from bulk parallel to it.^99^ Rotation is usually slowed by a factor of 2‐6,^93^ but at times even for several orders of magnitude.^99^ This is presumably focused on sites of large charge density^98^ and can extend to about 8.5 Å or 3 to 4 layers.^13^ To sum up, in general, less than 5 layers are perturbed by a factor of less than 5, following a broad power law distribution according to the distance.^93^

In relation to the small volume of a water molecule, most proteins represent a large excluded volume of comparable magnitude. Additionally, most globular proteins show a similar energetic surface disorder, so water dynamics around different proteins have been found similar overall, independent of protein size or function.^98^, ^100^ The slowdown is difficult to model by a single analytical function such as a power‐law^86^ though, because of the large chemical and spatial heterogeneity involved. Experiments can usually not provide detailed, site resolved mapping to resolve conflicting results, so this knowledge comes from computer simulations.

Locally, protein surface roughness has the highest impact on water dynamics in simulations, followed by conformational flexibility, since both change the excluded volume.^100^ Water in a concave environment experiences the largest confinement and thus potentially the strongest slowdown. Additionally, conformational fluctuations of the protein change the excluded volume and thus water residence times more strongly there than in sites of convex curvature. The reduction in average accessible volume that flexibility causes is well illustrated by the lower hydration of apolar protein cavities as compared with rigid model cavities, for example, in buckyballs.^101^ As a side note, H‐bonded water molecules in concave protein sites (clefts and pockets) are mainly bound to acceptor, not donor moieties.^100^, ^102^

The H‐bond lifetime, which in bulk is on average 2 ps,^13^ increases to dozens or hundreds of picoseconds on the surface, up to nanoseconds for surface clefts, and can reach up to microseconds and even milliseconds for water molecules buried in a protein.^93^ This gives an estimate of the speed of partner exchange between bulk and bound water and might be used to infer ligand transport. Since volume and surface change differently with radius, proportionally more buried and completely arrested water molecules exist in larger complexes.^102^, ^103^ These can usually not be distinguished from surface water in experiments.^13^ Simulations show that the degree of retardation differs in the order bulk < surface < interstice < bridging water,^104^ which is quite intuitive. Interstitial water resides between two biomolecular surfaces but is not H‐bonded to both. Bridging waters are H‐bonded between at least two different biomolecular sites. This can involve ligand‐protein, protein‐protein, or even intraprotein contacts. The longer the H‐bond lifetime, the longer the residence time, and the more ordered a water appears in structure determination methods.

#### 2.1.3 Protein mobility in water

Water and protein are thermally and mechanically coupled, which can be observed over a large range of length and time scales. It can be helpful for an understanding of protein motion to investigate its coupling to the solvent. It bears repeating, however, that whatever the influence of water might be, the functionality is part of the protein. Due to their higher connectivity and density, protein response to thermal energy is more akin to a solid than a liquid.^105^ Proteins change over the grand time scale of evolution, which is something water obviously cannot. Proteins “enslave” water as a mobile, mobilizing, and exchangeable workforce, a substitute functional group that is free of charge for the cell's metabolism. It is not straightforward to deconstruct all of water's multiple simultaneous services into macroscopic categories.

One mechanical function is lubrication, which includes dielectric screening, so water changes the protein energy landscape. Another is plastification. Those proteins that do not lose their structure fully upon dehydration show a similar loss of dynamics under dehydration as under low‐temperature conditions.^4^ The minimum hydration level needed for function is very close to the amount needed to form a protein‐spanning correlated H‐bond network, as opposed to individual water clusters that are oriented solely according to protein charges.^99^ The network character of water must apparently be activated so it can fulfil its lubricant role. Due to water's comparatively higher mobility and heat capacity, it enables and amplifies fast picosecond fluctuations at ambient temperature\ but suppresses them at lower temperatures by forming a frozen shell.^89^

Obviously, the water networks around proteins may be strongly influenced by cosolvents, such as ions. A well‐known example is the Hofmeister series,^106^ for which it was shown that ions affect both the water network as well as directly influencing the protein structure and dynamics.^107^, ^108^ The view of “slaved dynamics” holds that protein motion is governed by water motion, since the translation dynamics and H‐bond lifetimes of bulk water decide on the motion possibilities of the solvated protein:^109^ In simulations, the mechanical interaction ensures that protein dynamics are strongly affected by hydration water temperature, even if protein and solvent are thermostatted independently.^110^ Experimentally, the mobility of protein atoms under hydrated conditions increases sharply above ~220 K (‐50°C) but not under dehydrated conditions. The mean square displacement changes from < 1 Å^2^, which represents a glass‐ or solid‐like picosecond‐scale vibration involving energy barriers of 2‐5 kJ mol^‐1^, to 1‐3 Å^2^, which represents a rubber‐ or liquid‐like low nanosecond‐scale localized diffusion with an activation energy of 10‐20 kJ mol^‐1^.^89^ This dynamic transition temperature marks the onset of motions, for example, the hinge motion of lysozyme,^99^ that are a prerequisite for enzymatic activity.

Even in conditions of ample solvation at room temperature, most of a protein's dynamics are determined, that is, limited, by the viscosity of the solvent.^109^ This concerns local mobility in the form of, for example, side chain motion, as well as far‐range mobility in the form of diffusion. The first affects a first‐order reaction such as self‐cleavage upon conformational change, while the second affects a second‐order reaction according to collision theory. The maximum turnover rate of an enzyme, or any catalyst in aqueous solution for that matter, is around a million substrate molecules per second, the physical limit of mass transport through water by diffusion. Chemical processes could in theory operate faster than that, for example, in the gas phase. However, the usual turnover rate of an enzyme is rather in the range of about a thousand per second. This rate is governed mainly by internal motions.

All protein motions up to the major backbone rearrangements at the microsecond scale or longer are coupled to, and sensitive to, hydration.^89^ The longer the time scale, however, the weaker the correlation with the fast motions of water appears, so the large conformational changes and domain movements with activation energies of ~50‐100 kJ/mol^89^ that are often of interest seem more independent than the fast “rattling motions” of side chains. The only exception among fast motions are methyl group (rotation) dynamics, which are still active at very low temperatures and hydration levels,^99^, ^111^ and not coupled to supercooled solvent.^98^ This apparently liberates a protein's dynamics somewhat from its dependence on water compared with DNA or RNA.^112^

Sensitivity to hydration dynamics is also different between structures: A stiff β‐barrel is more affected at low temperature and low hydration, than a flexible α‐helical globular protein.^98^ Thus, the three‐dimensional build plays at least as important a role in protein dynamics as the coupling to solvent does. Proteins usually have a hydrophobic core, and their residues are less exposed to solvent compared with DNA or RNA. Among these biopolymers, proteins show the weakest temperature dependence and the fastest dynamics, followed by RNA, and then DNA as the slowest biopolymer.^113^ In addition, their respective hydration water shows differences in dynamics in the same descending order. Similarly, proteins with artificially arrested dynamics show slower hydration water dynamics too.^99^ Thus, the “slaving” or coupling is clearly mutual.^93^, ^99^, ^113^

It is chemically intuitive that hydrophobic groups, especially small ones such as methyl, will not couple to solvent to the same extent as hydrophilic ones do. Methyl groups (occurring in alanine, valine, leucine, isoleucine, threonine, and methionine) may sometimes partially take over the plastifying and lubricating roles of water.^89^, ^113^ It is interesting to note that many antifreeze proteins contain large amounts of valine, leucine, and isoleucine,^114^ and about twice the amount of alanine and threonine as an average protein.^115^ However, the influence of methyl group dynamics on the folding, flexibility, or antifreeze activity of these proteins, for example, by arresting their mobility in a simulation, has apparently yet to be tested.

### 2.2 Water in proteins

#### 2.2.1 Occurrence of crystallographic water within proteins

Water with such slow dynamics that it can be resolved in a crystal structure can be considered an extension of the protein.^93^, ^116^ Like for protein residues, the functional importance can vary, and the correct assignment of crystallographic water can be challenging.

A resolution of 2 Å is necessary to resolve water molecules, and there is a validated crystallographer's rule‐of‐thumb of a total of 'one water per protein residue' at this level.^117^ At 1 Å resolution, approximately 66% more water molecules are resolved than at 2 Å, but the increase is mostly due to surface waters.^117^ At a resolution of 1.5 Å and better, a continuous hydration layer at the protein surface can be observed.^118^ The detection of water in the interstice between two proteins also depends on resolution, but not as strongly, and interestingly, the detection of water between a ligand and the protein hardly depends on it at all, at least not at resolutions better than 2 Å.^119^

Crystal data are nowadays usually not collected at room temperature, but at ~100 K to minimize damage to the proteins in the intense synchrotron beams. The crystallographic temperature or *B* factor is a measure of uncertainty around a mean position and is influenced by several variables.^118^ Due to reduced thermal fluctuations, at 100 K about 1.5 to 3 times more water molecules are resolved in crystal structures than at room temperature.^120^ One explanation for uncertainty in the position of an atom is the amount of mobility it has. The *B* factor can be related to the root‐mean‐squared fluctuations (RMSF) of an atom.^121^ However, disorder in the crystal matrix can also lead to higher *B* factors, eg, when two water molecules occasionally occupy an active site, but a single solvation site is assigned. Depending on how the *B* factor are assigned, they have been found to linearly correlate with the crystallographic occupancy.^122^

The *B* factor declines steeply with the number of H‐bonds a water molecule makes to the rigid protein structure in its vicinity.^119^ The first established bond has the highest impact, with successive bonds causing less reduction in mobility. After the third^116^ or fourth^119^ bond, there is no significant difference anymore, even if there are more H‐bonding possibilities (polar contacts) available. At a given excluded volume, the residence time of a water molecule therefore depends strongly on the number of polar protein contacts it has.^123^, ^124^ A large cavity lined with hydrophobic residues that looks empty to crystallography can still in principle be found solvated by NMR,^125^ though this has to be interpreted with caution.^123^ Polar contacts with other water molecules trapped in a large hydrophobic cavity do little to slow down dynamics,^121^ since these waters are similarly mobile, and are both donors and acceptors.^86^

Water on the protein surface or between two proteins is on average only connected by one H‐bond, while water at the interface between a ligand and a protein is on average connected to the complex via three H‐bonds.^119^ Water with only one or two H‐bonds to a complex still has a higher *B* factor than the average protein atom.^119^ Water buried in the interior of a protein was found to establish three,^102^ four,^103^ or five^124^ H‐bonds. This can exceed the bulk value of 3.5,^22^, ^44^ since the polar protein contacts that are usually counted are not limited to water's spacious tetrahedral H‐bonding network geometry. The term “buried” usually means that a molecular entity's SASA is ≤5% of its maximum possible value. The *B* factor of such buried water with three or four bonds is even lower than the structural average of the protein,^119^ presumably because such waters bridge different secondary structure elements.^116^ Importantly, buried water with a low *B* factor tends to be conserved in the crystal structures of evolutionarily related proteins.^102^, ^116^

It is interesting that the *B* factor relation also holds vice versa: Protein atoms that H‐bond exclusively to a buried water show a lower *B* factor than when exclusively H‐bonding to another protein atom.^116^ This observation is valid for all protein atoms but concerns mainly the protein backbone amide nitrogen since it can form only one H‐bond, while the carbonyl oxygen can form two simultaneously. Amino acid side chains buried in the protein core are mainly hydrophobic, so the majority of buried water molecules forms H‐bonds to the protein backbone.^116^

Water is used by proteins to satisfy H‐bond needs that would otherwise be left unmet after the hydrophobically driven folding process is completed. Consequently, backbone H‐bonds that are formed exclusively with water and not to other protein residues are commonly found for regions that are neither involved in helical nor sheet conformation.^116^ Whether exclusively bonded or not, water is more rarely observed in α‐helices than in β‐sheets, and most often in coil regions.^103^, ^116^ The difference between α‐helices and β‐sheets is not caused by the number of cavities that could host water, since that is largely independent of secondary structure.^102^ It is also not a consequence of overall hydrophobicity, since β‐sheets are in general more hydrophobic than α‐helices. Instead, it is likely due to secondary structure flexibility and thus residence times.

In general, buried polar side chains are flexible or evolutionarily optimized enough to find other protein atoms to bond to and do not rely on water in the same way that main chain atoms do.^116^ As would be expected, charged and polar moieties like arginine and glutamic acid are also the main hydrated side chains in protein‐ligand interfaces.^119^ Lysine, as a charged amino acid, is often hydrated in rigid protein‐ligand interfaces,^119^ but when it lines a spacious cavity, it can assume different rotameric states with ease. It is often found disordered in crystal structures and also imparts this disorder on nearby water.^101^, ^124^, ^126^, ^127^ The aromatic moieties tyrosine and tryptophan are more hydrated than is generally assumed.^119^

In unliganded protein crystal structures, glycine has the lowest hydration propensity among the amino acids,^103^ presumably because of its large flexibility and small size. This is reversed in the rigid interfaces of protein‐ligand complexes, where it has the highest main chain hydration propensity, comparable to the serine side chain.^119^ The reason probably is that its backbone is more accessible than the backbone of any other amino acid. Proline's amide nitrogen, on the other hand, does not even form H‐bonds,^116^ yet is remarkably frequently observed next to buried water in unliganded crystal structures.^103^ This is probably due to its rigidity, which decreases the *B* factor in its vicinity. Interestingly, this is contrasted by proline in protein‐ligand complexes where it has the lowest hydration propensity among all side chains.^119^ The two special amino acids glycine and proline thus behave as opposites in a way. It could be posed that proline belongs to the nonpolar amino acids with regard to water that gets displaced easily upon ligand binding. A survey of crystallographic water next to proline and its *B* factors might be informative for this but apparently has not been conducted to date.

Buried water molecules can be found at all distances from the protein surface, but the overwhelming majority is found at less than 3 Å, or only about one protein atom depth.^102^, ^103^ Roughly 60% of buried waters are found “alone,” 20% occur as a cluster of two molecules, 8% as a cluster of 3 molecules, and so forth.^102^, ^103^ Each additional water molecule beyond the first only forms about 1 to 1.5 additional H‐bonds to the protein, while the remainder is formed with the other water molecules.^103^ The overwhelming majority of the clusters assumes a linear geometry, as observed especially for four‐membered clusters where many alternative configurations would exist.^103^ This is different from the ideal hydrophobic or vacuum situation, where quasi‐planar cyclic or compact clusters are found to be more stable (see Section 1.1).^128^

#### 2.2.2 Wires and charges

The linear water wires that are found dispersed throughout proteins are currently hypothesized to serve the function of an internal water exchange.^11^ It was previously assumed that buried water can only escape to the bulk with the help of rare and large‐scale protein fluctuations, such as unfolding.^93^ However, the analysis^11^ of one of the longest simulations that have been done to date, a millisecond all‐atom trajectory of bovine pancreatic trypsin inhibitor in explicit water,^129^ showed that access to the protein interior can occur through a rare event that requires no dramatic changes in protein structure: A linear chain of water molecules forms transiently (<5 ns) and repeatedly. This “flushing,” which at times may drag a ligand with it, has been observed for several proteins and has been dubbed “aqueduct mechanism.” In some cases, like myoglobin,^130^ some cytochrome P450's,^131^ or the green fluorescent protein (GFP),^132^ it can link preexisting water chain segments.

A further important point is the use of water as a conductor.^6^ Protons and hydroxide anions travel faster in neat water than particle diffusion would allow.^133^ This is mediated via the Grotthuss mechanism:^134^ The electronic charge or charge defect, rather than the proton itself, moves via a series of breaking and reforming covalent and H‐bonds. Several proteins involved in energy transfer like GFP,^132^, ^135^ photosystem II,^136^ or cytochrome f^137^, ^138^ have evolved to make use of this and employ water, usually interspersed between amino acids as short linear water clusters or “proton wires” to transfer charges. The maximum of 5 water molecules between donor and acceptor group in the high dielectric medium of neat water (ε ≈ 78)^139^ is surpassed by proteins through the use of easily ionizable side chains (low pK_a_), and through their more vacuum‐like internal dielectric constant (ε ≈ 4), which lowers the activation energy of proton transfer by ~4 kJ mol^‐1^ compared with bulk water.^140^ It has been proposed that water is prevalently used for ion transfer in biological systems because it is more mobile than amino acid residues, has a greater dielectric response, and is not subject to random mutations.^141^

Electric signal transduction occurs not only within proteins but also across the cell membrane. Because a membrane has ~30 Å thickness, pore proteins such as aquaporin, gramicidin A, or the various ion channels usually contain ~10 water molecules in the water wire crossing the pore region. Since membrane proteins are difficult to crystallize and resolve, and the pore walls are lined with many hydrophobic residues, these water molecules are often too dynamic and either partially or fully absent from crystal structures,^142^, ^143^ but they can be confirmed by simulations.^144^, ^145^, ^146^ Even though these proteins all feature a narrow cylindrical single‐file water region, due to unique and sophisticated amino acid placement, they interact with water in different, distinct ways.^147^ Aquaporin excludes ions efficiently, yet gramicidin A^148^ and the ion channels conduct them efficiently, even though all three show similar bulk‐like water diffusion through the pore.^141^ Hydrophobic protein pores can act like carbon nanotubes in this regard,^149^ conducting protons even faster than in bulk, since the internal water wire is prearranged by cooperative water‐water H‐bonding for one‐dimensional Grotthuss‐type transfer.^150^ Hydrophilic pores, on the other hand, have a chance to exclude ions of positive or negative charge by placing amino acid residues of the same charge in a narrow space (the selectivity filter). In aquaporins, this is enhanced by the mirror symmetry of the pore and the opposing macrodipoles of two hemi‐helices.^143^, ^151^ This forces the water molecules to flip their dipole in the wire centre during transport and prevents their reorientation. The push‐and‐pull on the H‐bonded half‐chains is facilitated by side chain motions, which pump water through the constricted area.^152^ Polar residues serve as H‐bond anchors; together, this repeatedly interrupts the H‐bonded chain.^153^ All this, it seems, ultimately prevents the Grotthuss mechanism from functioning in the aquaporin protein family.

#### 2.2.3 Cavity solvation

There are a few examples of proteins whose intimate connection to water is evident, such as the aquaporins. The tunnel this protein forms through the cell membrane is not representative for a classical cave‐like binding site however. It allows for almost unrestricted water flow and does not host a ligand (excluding channel‐blocking agents). In contrast, a typical small molecule binding site hosts only a small cluster of secluded and dynamically retarded water molecules.

The common observation that a cavity without ligand does not just remain empty is colloquially known as *horror vacui*, “nature abhors a vacuum.” In general, the larger the cavity, the higher the probability to find it hydrated.^102^ An estimated 90% of crystal structures in the PDB feature some form of buried water molecules.^103^ Of course, not all of these can be equally important: Less than 2% of resolved water in protein‐ligand complexes with measured binding affinities is in contact with the ligand.^119^ These few, however, can influence ligand recognition.

The van der Waals volume of a single water molecule is 12 Å^3^, but due to the spacious H‐bond network, it occupies 30 Å^3^ in the bulk phase. In the dense distorted water layer at the protein surface, it has an average volume of ~25 Å^3^ (a 20% reduction, with a wide distribution of values), and in the interior of a protein, this goes down to 23 Å^3^ (with a small distribution of values).^154^ If the size of a cavity is known, it is thus relatively straightforward to estimate the maximum possible number of water molecules it can host. The actual number, however, is very hard to estimate.^123^ This is due to several factors: One is that there are different ways to define a cavity, and multiple methods to calculate its size.^155^ Another is that proteins are dynamic entities: Size, solvent accessibility, and even surface hydrophobicity of a cavity can and usually will change due to fluctuations.^156^

Cavities large enough to host water typically comprise only ~1% of the protein volume.^102^ The number of observed cavities decreases roughly exponentially with increasing cavity size, so there are many more small than large, and many more empty than solvated cavities.^102^ The possible maximum size of a cavity increases mainly with protein size.^102^, ^116^ The upper limit found for a buried cluster seems to be around 20‐30 water molecules or ~650 Å^3^ (as outlined by the Conolly surface method),^156^ probably because more would reduce the number of intra‐protein contacts and destabilize the secondary structure.^102^, ^155^ Common hydrated cavities have a size below 350 Å^3^ and host less than 15 water molecules.^156^

In large pockets lined with polar residues, water can exist in compact arrangements other than the previously described, common linear geometry. These clusters are anchored by polar residues and structured by the field of the protein. They can, but often do not, resemble the compact minimum energy configurations of vacuum or gas phase simulations (see Section 1.1). The mutually H‐bonded clusters that are most stable in vacuum are understandably observed mainly in large apolar pockets.^121^, ^128^, ^157^, ^158^

In principle, very large apolar pockets can be hydrated as well, even if the occupying water molecules are too mobile to be easily resolved by regular crystallographic means.^159^ Several factors work against this, though. One is that truly nonpolar cavities are very hard to find, even if engineered, and become increasingly unlikely with cavity size.^155^, ^156^ Additionally, because proteins “breathe,” polar residues will sometimes move to the cavity surface and “draw out” residing water molecules.^156^

Another is that almost all nonpolar cavities have a size well below ~40‐60 Å^3^.^156^ Additionally, these cavities change their size over time and are thus effectively smaller than they seem in a crystallographic picture. This means that usually not more than one or two water molecules can be accommodated. The confinement in a space less than 40 Å^3^ should limit the mobility or respectively the RMSF of a water molecule sufficiently to resolve it in regular crystallography.^123^ Experimentally, there is conflicting evidence, with somewhat more support for empty cavities.

Recent consensus from most calculations is that transfer of water from the bulk into fully hydrophobic cavities, such as modeled by the interior of a buckyball,^160^ is energetically favourable but incurs such a large entropic penalty that the overall free energy of hydration is at best around zero.^123^ Confined waters form ordered, mutually H‐bonded clusters.^128^ Only when a cluster of at least 3 to 4 water molecules can be accommodated at once, then the free energy of solvation can become favourable.^128^, ^158^ Perhaps because of this “crossover” of energetic considerations with cavity and buried cluster size distribution, the water tetramer seems to be relatively frequently investigated in hydrophobic protein cavities.^121^, ^128^, ^157^, ^158^

Most cavities feature a surface with mixed areas, though. Due to their partly nonpolar character, the average water density within a cavity is often found to be lower than in bulk water, reduced by ~20%,^131^ 50%,^159^ or more. This is in line with the decrease in water density in the immediate vicinity of hydrophobic solutes.^82^ Thus, the 15% increase in water density at the (mostly hydrophilic) protein surface cannot be extrapolated to estimate cavity solvation. These differences are the reason why solvation algorithms for molecular simulations, which usually only know about bulk water density, or at best about surface‐exposed groups, generally fail to adjust to the inhomogeneous requirements for solvation in protein cavities. The same problem holds in reverse: The fact that bulk water has not been modelled successfully using water clusters in (vacuum) confinement, points to the large differences in the description of the two “water species” (see Section 1.2).

## 3 LIGANDS AND WATER

### 3.1 Binding site solvation

#### 3.1.1 Occurrence of interfacial and bridging waters

Important water molecules seem rare if compared with the crystallographic total, but they are actually frequently observed when looking only at the space around ligands. For example, there is a subset of ~400 structures in the PDB that were solved at room temperature with a resolution ≤2 Å, which also include water, a ligand, and binding affinity data, collected in the PDBbind database.^161^ In it, more than 80% of the complexes show at least one water molecule involved in the protein‐ligand interface.^119^ Of these, about half has some access to bulk solvent, and half can be classified as buried. In contrast to the majority of buried waters in unliganded proteins, where the backbone is the main hydration site,^116^ the subpopulation of water molecules between protein and ligand is in ~60% of the cases found on the protein side chains.^119^ This is probably because ligands are expected to bind to side chains more frequently than to the backbone due to the side chain's larger surfaces, and greater range of functionality, which ensures specificity.

Two thirds of all polar interactions of interfacial water molecules are made with the protein, and only one third is made with the ligand.^119^ This is likely just because of the larger volume a protein occupies. Importantly, of all interfacial water molecules, ~80% are directly bridging between the ligand and the protein.^119^ In contrast to protein‐ligand complexes, only ~30% of all interfacial water molecules in protein‐protein complexes are bridging between both entities.^162^ The likely reason is that due to bulkiness and steric constraints, two proteins simply cannot get as close to one another as a protein and a small molecule can. Among direct polar protein‐protein contacts in complexes, water‐mediated interactions are at least as abundant as protein‐protein H‐bonds.^162^

Peptide ligands represent a borderline case since the ligand is still of moderate size but could be classified among protein‐protein interactions. For example, the oligopeptide‐binding protein A (OppA)^127^, ^163^ is among the ~6% found in the above‐mentioned PDBbind dataset that includes more than eight bridging water molecules in a ligand‐occupied binding site.^119^ Ligands with such a large amount of water crystallized in its vicinity are expectably polar and of intermediate size. Though therapeutic peptides and proteins are of increasing pharmaceutical interest, the common notion is that drug‐like molecules are small and nonpolar, as stated in the widely known Lipinski's rule of five.^164^ For the typical small compounds with low polarity in drug design studies, one can thus expect that significantly less than 8, but usually 1 or more water molecules, have to be examined; the average has been estimated at 4.5.^119^

The enthalpic components of water confinement depend on the chemical nature of the container. By itself, only the fluorine atom is appreciably more electronegative than the oxygen atom, so this case is usually irrelevant in the context of biomolecules. The mass of a protein consists roughly of 55% carbon, 20% oxygen, and 15% nitrogen.^165^ The interaction with carbon is relatively weak due to its low polarity. A priori, it could be postulated that the potential energy of interaction offered by a protein oxygen is not much different from the one of a water oxygen. Nitrogen is less electronegative and might offer weaker bonds; however, it also often bears a net charge. Overall, the chemical identity and geometric arrangement of typical surrounding protein and ligand atoms will likely lead to weaker polarization and less ideal H‐bonds than in bulk water. With the enthalpic contribution to water association being either small, unclear, or perhaps roughly equal between bulk water and protein environments, entropy is bound to play a large role in water confinement.

As mentioned, water at the protein‐ligand interface establishes on average 3 H‐bonds.^119^ This is slightly less than the ~3.5 H‐bonds in the bulk phase. One can therefore expect a slightly lower polarity of such interfacial water molecules compared with the bulk or surface, due to reduced charge shifts through H‐bonds. To the best of our knowledge, the only study that attempted to extract the dipole moments of binding site water molecules directly from crystallographic protein structures has been conducted on the OppA protein.^166^

OppA's binding site water molecules are highly conserved. They have *B* factors that are even slightly lower than the protein average, indicative^119^ of four or more polar contacts to the surrounding protein. While a low *B* factor might simply stem from confinement, the chemical nature of the confinement could change the trapped molecule's polarization. An dipole moment of 2.5 D was estimated by a study on 14 crystal structures on OppA^166^ that optimized the prediction of the Dowser solvation algorithm^167^, ^168^ to recover OppA's resolved binding site waters. This dipole moment value would in turn be indicative of only about 3 H‐bonds and should be compared with 1.8 D in the gas phase, and 3 to 3.5 D in the bulk phase (see Section 1.1). While the 2.5 D estimate was not derived from rigorous QM calculations but from simpler atomistic considerations, it was consistent with another estimate derived from the solvation free energy of a point dipole in a dielectric continuum.

It has been argued that due to the lowered dipole moment, ligand‐bound water molecules might not be such an important source of polarity in a binding site after all.^101^ Whatever the local environment, the average electric field in the interior of proteins and membranes is much weaker than in bulk water. Proteins typically are modelled with a dielectric constant of 4 in continuum approaches. In biomolecular simulations, water models with appropriately lowered polarity have been shown to penetrate more easily into lipid bilayers.^169^ This should also be the case for hydrophobic protein areas, where these differences matter most. Because of their high mobility, internal water molecules can respond more strongly to an electric field than proteins can. They thereby contribute to the value of the actual dielectric constant inside proteins, and change the pK_a_ value of ionizable groups.^124^

As the polarizability of a water molecule decreases with increasing coordination number due to charge flow through H‐bonds, it becomes less responsive to the additional electric field of a potential incoming polar ligand. It can be argued that isolated, non‐H‐bonded water molecules in a hydrophobic protein binding pocket might be more reactive and amenable to CT towards a ligand than bulk water molecules are. They might therefore be an important source not of polarity, but of charge density. CT may have a nonnegligible impact on the charge distribution and therefore reactivity of a ligand. The arrangement of water molecules surrounding a ligand in a binding site may favour either reactant or product formation or may shift the balance between several possible products.

A recent example in this regard is a study on tubulin, a protein involved in cell division and microtubule formation.^104^ The small molecule inhibitor TN16 can bind to tubulin in several different poses. Two static water molecules have been found to bridge the interactions between ligand and protein and induce a shift in electron density from TN16 to tubulin. This charge relay stabilizes certain binding modes of this inhibitor and also helps to discriminate it from other active ligands. Examples like this, where specific water molecules make a pharmacological difference, are increasingly recognized.^6^, ^170^

### 3.2 The binding process

#### 3.2.1 Complex formation

At low concentrations, two molecules of interest translate and rotate freely without encountering each other. Once they collide, they associate only if their mutual attraction (potential energy) is more pronounced than their mobility (kinetic energy); in other words, if the favourable binding enthalpy overcomes the unfavorable loss of translational and rotational entropy. At higher temperatures, collision frequency increases but so does the kinetic energy that has to be overcome. In solution, the strong interactions between solvent molecules may additionally favour the complexed state through the hydrophobic effect.

Upon association, the translational and rotational degrees of freedom of protein and ligand turn into the mutually restricted ones of the complex; additionally, vibrational degrees of freedom appear between the molecules. The kinetic energy redistributes over the new degrees of freedom, and the residual motion can be estimated from the vibrational entropy of the complex.^171^, ^172^ The more residual motion the complex has the more the loss of translational and rotational freedom upon binding is counteracted. This residual vibration decreases as the interaction strength of the complex increases, and the vibrational degrees of freedom stiffen.

The loss of mobility as a complex tightens is the cause of one variant of the so‐called enthalpy‐entropy compensation phenomenon, described by the competing signs of the ∆*H* and *T*∆*S* terms in the Gibbs equation. It appears to be a property of basically all intermolecular (that is, weak) association, of which H‐bonding in aqueous solution is the most frequently encountered one.^171^, ^172^, ^173^, ^174^ Both *H* and *S* depend on the preferential distribution of the system among the lower or higher energy levels. The amount of energy compensation is limited by the following: (a) the entropy loss that represents complete immobilization of the molecule. A rigid ligand design minimizes this entropic loss and (b) the enthalpic gain of all new (noncovalent) interactions. The sum of these contributions must overcome the sum of any competing solvent‐solute interactions.

#### 3.2.2 Competition

The restricted accessibility of an active site can be necessary to prevent strong or indiscriminate binding of other encountered molecules in the crowded cellular environment. It also hinders the approaching “intended” ligand, so the equilibrium between binding and dissociation can be slow to attain experimentally, which is a potential source of error. Once a ligand reaches the active site, it has to compete with resident water molecules to bind successfully.

Conceptually, energy has to be invested first in the displacement process to pull the water molecule out of a specific site it is bonded to. This desolvation cost must always be unfavorable as long as water is held there somehow,^175^ but the strength of the association is limited by the maximum strength of an H‐bond (30 kJ mol^‐1^) and is likely below the average value of bulk water H‐bonds for an uncharged protein site (see Section 1.1).

Water that has weak and nondirectional van der Waals contacts with a hydrophobic environment enjoys little enthalpic stabilization, is weakly polarized, and will be easy to remove; water that has many directional, immobile polar contacts enjoys much enthalpic stabilization, is strongly polarized, and will be hard to remove.^176^ The gas/vapour phase and hydrophobic confinement have a similar effect on polarization and show a roughly similar partitioning between these phases and the liquid bulk phase. A terminology of “hot” waters, which are mobile and easy to replace, versus “cold,” ie, ice‐like waters, which are tightly held and hard to replace, has recently been proposed.^6^

In the long run, a successfully removed water becomes one with the bulk. The liberation from a confining excluded volume with limited motion possibilities to a mobile and spacious environment means a large increase in accessible microstates for the water molecule—an entropic gain. Hydrophobic association is experimentally recognized by a dominating entropic component, as seen in isothermal titration calorimetry (ITC) ligand binding measurements for example. Water in a hydrophobic enclosure with just a few order‐inducing H‐bonds has been identified as the most advantageous target motif to increase the entropic contribution to binding in ligand design.^66^ The maximum amount of entropy that can be expected from a single, completely immobilized water molecule was estimated at up to 8 kJ mol^‐1^ at room temperature.^87^

The displacement of a water molecule from an active site in favour of a newly added interaction point is only favourable if the enthalpic penalty of breaking the H‐bond of the water molecule is outweighed by the gain in ligand‐protein enthalpy and the gain in solvent enthalpy and entropy. This is a more complex variant of enthalpy‐entropy compensation, and a ubiquitous obstacle to the achievement of a large binding free energy in ligand design.^177^

The chemical identity of the ligand and the displacement of water during binding add to the overall ∆*H* and *T*∆*S* terms in a binding process such that in a homologous series of ligands, often only small changes in ∆*G* are observed despite large changes in both ∆*H* and *T*∆*S*. The series of ligands that binds to the OppA protein^127^, ^178^ is a well‐illustrated example: ∆∆*H* and *T*∆∆*S* of the modified ligand and the displaced, previously bound water molecules all sum up to give small overall changes in binding affinity, which cannot be correlated with the simple contact surface area between protein and ligand anymore.^179^, ^180^ In the same way, if two homologous ligands experimentally show a similar enthalpic and entropic contribution to binding, for example, in ITC measurements, this does not guarantee a preservation of the binding mode, but it might be caused by competing effects.

However, molecules which are mainly hydrophobic can still bind with an enthalpy‐driven signature. This has been somewhat puzzling several times in literature.^66^, ^181^, ^182^, ^183^ A likely explanation is that a protein cavity can be empty (or poorly solvated) to begin with. In these cases, the desolvation cost has to be paid only (or mainly) for the ligand. Any new interactions formed in the cavity are an “enthalpic bonus” in favour of the ligand bound state. No water molecules are liberated, so there is no entropic gain. In the mouse urinary protein I for example, the association of a hydrophobic ligand to a protein cavity shows a dominating enthalpic component.^183^ In short, ligand binding to a dry hydrophobic enclosure is currently believed to be the most advantageous target motif to increase the enthalpic contribution to binding in ligand design.

### 3.3 Ligands

#### 3.3.1 Ligand design

The entropic contribution to ligand binding by water liberation becomes proportionally larger when several water molecules are displaced at once, such as in the case of Streptavidin. The binding of biotin to this protein is extremely strong and specific, one of the strongest known noncovalent associations in nature.^184^ Its entropy‐driven signature originates partly from the displacement of an ordered cluster of five^66^, ^185^ to seven^186^ water molecules in the mainly hydrophobic, enclosed binding site.

A suspenseful example in a nonclassical binding site involves the proton‐selective M2 transmembrane channel in the viral envelope of the influenza virus. It can be blocked by adamantane‐based inhibitors that lock the channel in a nonconducting, closed state. The pore interior contains two stacked planar water tetramers, of which the upper one interacts with the amine‐moiety of the commonly used channel blocker. When mutant strains developed a resistance against the blocker, a more elongated drug variant was designed with the aid of MD simulations.^187^ It displaces the upper water tetramer completely and now interacts with the lower one. The drug thereby penetrates more deeply into the channel and bypasses the resistance‐inducing mutant site. The cluster of ordered water molecules increases in mobility, which increases the entropic contribution to ligand binding.^19^

In structure‐based or “rational” ligand design, a classical strategy is to increase the binding affinity of a ligand by replacing selected crystallographic water molecules with (additional) ligand atoms. It is usually simply assumed that a modified ligand binds in the same pose as the lead compound. If both an empty and a liganded protein crystal structure are available, the number of displaced water molecules can be counted directly and the binding process can be rationalized in hindsight. This is currently the status of most investigations. However, it would of course be preferable to know water displacement chances, resulting binding poses, and binding affinities, before actually synthesizing a potential ligand.^188^ Attempts to predict solvation patterns and their contribution to ligand binding have been ongoing for decades.^6^, ^170^, ^189^ In several instances, however, ligand design attempts have either failed to displace a resident water molecule^190^ or in the course of trying have worsened the binding affinity of the compound.^191^

#### 3.3.2 Specific ligand modifications

Water can donate and accept H‐bonds, acting simultaneously as a Lewis acid and a Lewis base. A functional group that has either of these abilities, such as a hydroxyl, carbonyl, amine, or thiol, can replace a specific water molecule. Water can additionally switch a Lewis acid to a Lewis base functionality, or vice versa, change its orientation, and extend it by almost 3 Å.^6^ If a water molecule has been identified as bridging between a polar ligand atom and a polar protein atom in a crystal structure, it can be targeted for replacement by adding chemical groups to the ligand. The ligand ideally is modified such that it mimics the bridge, in order not to lose enthalpic contributions favorable for binding. An atom with a similarly strong electronegativity as the water oxygen should be placed in a similar geometric position. This requires a high‐resolution knowledge of the binding site.

Often, a polar ligand atom is conceptually first changed to a carbon, which then receives a polar substituent, effectively “projecting” the original polar moiety outwards. Intuitively, a hydroxyl group (─OH) on that carbon would mimic water most closely. The C─O bond length of ~1.4 Å however is too short to project the oxygen atom far enough. A hydroxymethyl group (─CH_2_OH) projects the oxygen about 3 Å further, just slightly more than the average H‐bond length. This is well illustrated in a series of crystal structures of the OppA protein,^127^, ^163^ where the central glycine of a bound tripeptide ligand can be exchanged for serine. The serine oxygen takes almost the same geometric position as the water oxygen observed for the glycine‐containing ligand.

Another suitable choice that has been used successfully in the ligand design literature to replace water molecules^192^, ^193^ is the cyano group (─C≡N). It has slightly less molar mass than the hydroxymethyl group (a molar mass below 500 g mol^‐1^ is one of Lipinski's rules of five for orally active drugs^164^). The cyano group projects the polar atom forth for about 2.5 Å. Of course, no covalently bound substituent on a ligand will ever be able to follow protein fluctuations as flexibly as an H‐bonded water molecule. In this regard, the rigid cyano group is less adaptable to a flexible binding site than the rotatable hydroxymethyl group. Also, successful water replacement and ligand binding might rigidify the protein cavity and reduce its conformational fluctuations. This will be even more pronounced the more rigid the ligand is and will most likely add an unfavourable entropic cost to the binding process.

#### 3.3.3 The (computational) benchmark set of 2018

As it is not rare to find water molecules buried in an active site, both before or after binding, suitable proteins to explore the influence of water on binding free energy should be easy to come by. Recently, seven protein‐ligand and protein‐modified ligand pairs were proposed as a benchmark set.^175^ The set was intended to facilitate comparisons between computational methods. The proposed complexes fulfil certain criteria such as measured binding affinities, high‐resolution crystal structures, and buried water in the active site. Notably, only one or two water molecules and relatively small ligand modifications were considered in each case.

Four of these seven pairs compare ligand modifications in the heat shock protein of 90 kDa weight (HSP90).^192^ The other three proteins belong to the kinase,^194^ protease,^195^ and dehydratase^193^ protein classes; all examples in the set except the last one act on other proteins.

For one of the four ligand pairs bound to HSP90, the change between the pyrrolopyrimidine‐based ligands consisted of the replacement of a ring carbon atom with a nitrogen atom. This ligand atom is in contact with two conserved bridging water molecules, but not with the protein directly. Despite this, and the increase in ligand polarity, the experimental binding affinity decreased by two orders of magnitude.^192^ A calculation that employed inhomogeneous fluid solvation theory^196^, ^197^ estimated that the more polar ligand stabilizes the two water molecules in their position and leads to more favourable binding.^175^ Both the experimental^192^ and theoretical^175^ study assume a slight water rearrangement, but importantly, not a full displacement or a ligand binding mode change.

An immediate and very recent follow‐up investigation^198^ employed an alchemical calculation method.^199^ Additionally, an enhanced sampling methodology, a form of replica exchange, was used on the binding site. Through these measures, a more correct decrease in binding affinity by about one order of magnitude was now predicted. Though this represents a significant improvement, this result for the HSP90‐pyrrolopyrimidine complex still differs from the experimental value by ~13 kJ mol^‐1^. In general, several of the benchmark set test cases deviated by more than thermal noise.^198^ This might of course be either because the experimental value is incorrect, but it is also at least equally likely that this is due to simplifications that are common in ligand design, in this case also in the employed program suite. This case study highlights the need for powerful computational methods^200^ to tackle the deceptively simple problem of water‐governed ligand binding.

## 4 CONCLUSIONS

Water is considered a basic substance, but its ability to form hydrogen bonds makes it actually highly complex, and essential to molecular biology. The mobile charge density on the water oxygen causes the flexible nature of the hydrogen bond, which leads to a large range of possible values for a water molecule's polarity, polarizability, and volume. Water is one of the smallest possible molecules and highly mobile. Its potentially strong enthalpic interactions get distributed over a wide entropic range depending on both short and long range effects of the environment.

Water couples mechanically to the hydrophilic groups of a solute, among which protein surface residues are especially important. This coupling enables functional protein motion as long as water's dynamic network character is intact. For a protein solute, the malleable nature of water leads to a disordered, dense, and comparatively rigid solvation shell, but a dynamic and low‐density cavity solvation. Protein cavities are often empty, even if they are large, in contrast to the *horror vacui* idea. Water molecules become buried within protein confinement following specific patterns. Buried water with a low *B* factor tends to be evolutionarily conserved. It can be exploited by proteins to replace functional groups, add functionalities, and dynamically adjust the distance, connectivity, or strength of existing functionalities.

Vacuum represents ideal hydrophobic confinement. The structures and properties of water clusters in vacuum can be used to investigate similar clusters found in protein channels and binding pockets. Among these clusters, the linear tetramer and decamer, and the cyclic tetramer and pentamer draw most biological interest. The hexamer marks the transition to bulk‐like properties, but it is still insufficient to model the bulk phase.

The experimental inhomogeneity in water polarity, polarizability, and volume still complicates the computational prediction of binding pocket solvation, and its high mobility hinders convergence of ligand binding free‐energy calculations. Ligand design traditionally focuses on rather straightforward approaches. An increase of the enthalpic contribution to binding is attempted either through the increase of hydrophobic contact surface, or the addition of, eg, hydroxymethyl or cyano substituents to replace crystallographic water molecules. A small set of protein structures was recently proposed for benchmarking purposes in this regard.

The presence of “competitive” water that must first be displaced from a binding site causes a preceding desolvation cost in the binding process. Its enthalpic component is limited by the offered hydrogen bond strength of the interaction site. Weakly bound and polarized water is easily displaced. Strongly bound and polarized water that cannot be displaced acts as an extension of the protein and influences the ligand binding pose. The presence of adjacent water may increase ligand reactivity through polarization and CT. The majority of protein‐ligand complexes feature interfacial water molecules, most of them directly bridging between ligand and protein.

In contrast to the enthalpic contribution, water's entropic contribution to binding is potentially large and rather clear: It is limited by the complete arrest of water mobility in, and subsequent liberation from, singular confinement. For an empty binding pocket, there is no desolvation cost preceding the binding process, and no entropic contribution through liberation of confined water molecules into the bulk. The sums of enthalpic and entropic effects may compensate and results in a binding affinity that, even for a homologous series of ligands, must be interpreted with caution.

## CONFLICT OF INTEREST/AUTHOR CONTRIBUTIONS

The authors report no conflict of interest. M.M. has conducted initial literature research. M.M. and C.O. wrote the manuscript. Both authors have read and approved the final manuscript.
